# Predictive model of multiple emergency department visits among adults: analysis of the data from the National Survey of Drug Use and Health (NSDUH)

**DOI:** 10.1186/s12913-021-06221-w

**Published:** 2021-03-25

**Authors:** Georgiy Bobashev, Lauren Warren, Li-Tzy Wu

**Affiliations:** 1 RTI International, 3040 Cornwallis Rd., P.O. Box 12194, Research Triangle Park, NC 27709 USA; 2 Department of Psychiatry and Behavioral Sciences and Department of Medicine, Duke University School of Medicine, Box 3903, Durham, NC 27710 USA

**Keywords:** Emergency department admission, Predictive model, Random forest, Self-rated health, Mental health

## Abstract

**Background:**

In this methodological paper, we use a novel, predictive approach to examine how demographics, substance use, mental and other health indicators predict multiple visits (≥3) to emergency departments (ED) within a year.

**Methods:**

State-of-the-art predictive methods were used to evaluate predictive ability and factors predicting multiple visits to ED within a year and to identify factors that influenced the strength of the prediction. The analysis used public-use datasets from the 2015–2018 National Surveys on Drug Use and Health (NSDUH), which used the same questionnaire on the variables of interest. Analysis focused on adults aged ≥18 years. Several predictive models (regressions, trees, and random forests) were validated and compared on independent datasets.

**Results:**

Predictive ability on a test set for multiple ED visits (≥3 times within a year) measured as the area under the receiver operating characteristic (ROC) reached 0.8, which is good for a national survey. Models revealed consistency in predictive factors across the 4 survey years. The most influential variables for predicting ≥3 ED visits per year were fair/poor self-rated health, being nervous or restless/fidgety, having a lower income, asthma, heart condition/disease, having chronic obstructive pulmonary disease (COPD), nicotine dependence, African-American race, female sex, having diabetes, and being of younger age (18–20).

**Conclusions:**

The findings reveal the need to address behavioral and mental health contributors to ED visits and reinforce the importance of developing integrated care models in primary care settings to improve mental health for medically vulnerable patients. The presented modeling approach can be broadly applied to national and other large surveys.

**Supplementary Information:**

The online version contains supplementary material available at 10.1186/s12913-021-06221-w.

## Background

Although the emergency department (ED) provides a critical source of acute care, repeated ED visits constitute a major healthcare problem. Treatment received at an ED is considered suboptimal because it is not designed to provide continuity of care: it is very costly, and it contributes to overcrowding and provider/staff shortage [1, 2]. The cost of an ED visit is about 4 times higher than that of an office-based visit, which has a major impact on a nation’s healthcare system [3]. ED overcrowding is a key obstacle to healthcare delivery and places an enormous burden on the U.S. healthcare system [1]. It is important to identify predictors of individuals who are frequent ED users because they consume much more healthcare and represent a high-risk, medically vulnerable patient population [3]. Factors associated with having an inadequate timely access to primary care may be associated with ED use (e.g., lower education or income) [4, 5]. In addition, fair/poor self-rated health and severe or chronic medical conditions, such as diabetes, asthma, and cardiovascular or lung diseases, are associated with frequent ED use [5–7]. Substance use (e.g., overdose, injuries) and mental disorders also contribute to ED visits [7, 8].

Existing epidemiological research often has relied on traditional regression approaches (e.g., logistic regression) to estimate the *strength of an association* between a given factor and ED use, rather than the predictability of cases based on the knowledge of suspected determinants or risk factors. Perception of an independent variable (or a suspected risk factor) that doubles the outcome chances from 30 to 60% might differ considerably from the one that doubles the chances from 0.001 to 0.002%. In epidemiological research on association, it is common to find a long list of independent variables associated with an outcome. When a number of factors influence the outcome, it is not always clear if there are there some specific complex subgroups that stand out strongly enough to deserve special attention for target intervention.

Thus, the aims of this paper are to

1. evaluate and compare how well different models predict multiple ED visits,
2. validate the models across years, and
3. identify the most influential factors across multiple models.

We answer these questions by conducting predictive modeling. Predictive models estimate probability of an outcome for individuals with specific characteristics, and a number of models of different complexity can be used for making such predictions [9, 10]. Thus, the balance between complexity, interpretability, and consistency of predictors becomes an additional challenge [9–12]. A validated predictive model should not be taken as a test of a hypothesis or causal argument, but rather as a snapshot of what the population-level data show.

The National Survey on Drug Use and Health (NSDUH) is a valuable resource to study multiple visits to the ED. The large size of the NSDUH sample and the richness in measures (including substance use domains, mental health, self-rated health, medical conditions in the past year) allow us to develop high-quality predictive models. When a model captures the underlying relationships in the data, further increase in complexity would lead to fitting noise or “overfitting.” By using multiple years of the NSDUH dataset, we can validate *reproducibility* by using independent datasets for training, validation, and test purposes thus guarding against overfitting [13]. The approaches used in the study can be applied to other public health conditions.

## Methods

### Data source and sample

Data were from the public-use data file of the 2015–2018 NSDUH. The NSDUH is an ongoing, cross-sectional survey designed to provide national estimates of substance use in the U.S. [14–17]. The target population includes household residents from the 50 states and the District of Columbia (including shelters, rooming houses, and group homes; civilians residing on military bases). Participants are selected by representative multistage probability sample of respondents aged ≥12 years. Weighted response rates for household screening ranged from 73.3 to 79.9% over the 4 years and interviewing response rates ranged from 63.9 to 68.4% [14–17]. This study focused on adults aged ≥18 years using public-use de-identified datasets (*N* = 43,561 in 2015; *N* = 42,625 in 2016; N = 42,554 in 2017; N = 43,026 in 2018).

#### Data collection

Respondents were interviewed privately at their places of residence. Most sociodemographic questions are administered by interviewers using computer-assisted personal interviewing (CAPI)^1^. Other questions of a sensitive nature (substance use, health status) are administered with audio computer-assisted self-interviewing (ACASI), which provides respondents with a highly confidential means of responding to questions.

#### Dependent variable

Any ED use was defined as having one or more ED visits in the previous 12 months (“During the past 12 months, that is, since [DATEFILL], how many different times have you been treated in an emergency room for any reason?”). We focused on multiple ED visits (≥3 times/year) [18].

### Potential correlates of ED visits

Based on Andersen’s healthcare utilization model, we examined 46 variables that included predisposing (demographics), enabling (family income, educational level, population density of residence), and need-related (substance use, mental health, medical health, overall health) variables as potential determinants of ED visits [18, 19]. Thus, the variables selected into the study are not the result of a screening or data mining exercise but rather the result of careful selection based on prior literature and validated analyses.

#### Sociodemographics

Respondents’ age, sex, race/ethnicity, family income, insurance status, marital status, educational level, and population density of residence were examined as potential correlates of ED use [18, 20]. Race/ethnicity includes Non-Hispanic white, non-Hispanic African American/black, non-Hispanic “Other” and Hispanic. The non-Hispanic “Other” category includes Native American/Alaska Native, Native Hawaiian/Other Pacific Islander, Asian, and multiple-race.

#### Past-year substance use

Tobacco, alcohol, and other nine drug classes were assessed in separate sections, which included a description of the substance class and a list of substances in that class. Tobacco use included use of cigarettes, smokeless tobacco (i.e., snuff, dip, chewing tobacco, or “snus”), cigars, or pipe tobacco^2^. Illicit or nonmedical drug use included marijuana/hashish, cocaine/crack, heroin, hallucinogens, inhalants, prescription opioid pain relievers^3^, prescription stimulants/amphetamines, prescription tranquilizers, and prescription sedatives.

#### Past-year alcohol or drug use disorders

Respondents who reported alcohol or drug use in the past year were assessed by a set of structured and substance-specific questions designed to operationalize DSM-IV criteria for abuse of or dependence on each the substance class.

#### Past-month nicotine dependence

Nicotine dependence was defined as specified by the Nicotine Dependence Syndrome Scale (NDSS) and the Fagerstrom Test of Nicotine Dependence (FTND) [21, 22]. To optimize the number of respondents classified as having current nicotine dependence, NSDUH categorizes respondents as having nicotine dependence in the past month if they meet criteria for dependence as specified either by the NDSS or FTND [23].

#### Past-year mental health

Based on prior research on healthcare use, we examined major depressive disorder and anxiety characteristics [19]. Questions assessing major depressive episodes (MDE) were based on DSM-IV criteria [24]. Anxiety indicators utilized were “During the past [time period] (past 30 days or past 12 months, time period that respondent felt their worst emotionally), how often did you feel restless or fidgety?” and “During the past [time period] (past 30 days or past 12 months, time period that respondent felt their worst emotionally), how often did you feel restless or fidgety?”

#### Medical health

Medical conditions (asthma, chronic bronchitis, emphysema, or chronic obstructive pulmonary disease (COPD), cirrhosis of the liver, diabetes or sugar diabetes, any kind of heart condition or heart disease, Hepatitis B or C, high blood pressure, HIV/AIDS, cancer/malignancy of the larynx/windpipe or lung, or sexually transmitted disease) were assessed by a series of discrete questions. Asthma was defined for the current time period; sexually transmitted disease and heart conditions were defined for past year; and all other conditions were asked for the respondent’s lifetime.

#### Overall health

Respondents’ *self-rated overall health* had categories excellent, very good, good, fair, and poor. Fair/Poor health was associated with frequent healthcare use and chronic illness [6, 25, 26]. Following this analysis, we grouped fair, and poor categories to create a dichotomous indicator for self-rated health.

### Analysis methods

#### Training and validation datasets

We used two datasets for model building: one for training and one for validation. We included the results from two additional datasets to show how the models performed on earlier data. The training dataset (2017 data) was used to develop a model by performing model building and selection algorithms described below. Candidate models with similar performance on the training set were validated on a separate validation dataset (2018 data). Finally, the best performing model was rerun on several additional validation datasets from prior years (2015 data and 2016 data). In model selection, we selected the simpler and more interpretable model from similarly performing models (parsimony rule).

#### Full main effects logistic regression

The sample size is large enough to incorporate main effects from the entire set of 46 variables. However, the incorporation of all possible interactions is not feasible, and other methodologies should be used to identify potential interactions. Additionally, the predictive value of full regression is often not optimal because of the potential for fitting the noise.

#### Least Absolute Shrinkage and Selection Operator (LASSO) regression

LASSO regression methodology penalizes models for overfitting and controls for collinearity. Parameter estimates are generally “shrunk” toward zero, which guards against overfitting and allows for the identification of a robust set of predictors [27]. The estimation of standard errors for LASSO has been under development but there is no clear consensus on their interpretation [28]. This last restriction is not critical because the exact inference of the coefficient values is not an objective here.

#### Stepwise logistic regression (main effects)

Stepwise logistic regression was used to identify main effects associated with the outcome with an Akaike Information Criterion (AIC) to control for overfitting [13]. The actual values of the regression coefficient are not the objective, and for predictive purposes, stepwise regression can provide useful insights.

#### Classification tree

Possible interactions were estimated from a classification tree. Classification trees recursively partition the sample into groups where subjects within a group are more homogeneous than they are to those in other groups with respect to outcome [11, 13]. A tree model can be summarized in a single categorical variable with categories corresponding to the logical combination of variables defining the terminal nodes. This variable can then be added to a (stepwise) logistic regression to represent most prominent interactions.

#### Random forests

Random forests extend classification trees in two dimensions [11]. One dimension is to perform classification trees on each of many (e.g. 200–400) bootstrap samples from the original data. Each tree provides a prediction of individual outcome; thus, for each subject, we obtained an “ensemble” of predictions. The variance of these estimates across the trees characterizes prediction uncertainty. The second dimension is to choose only a random subset of predictors to be used at each sample partitioning. This additional use of randomization allows the model to incorporate useful, but weaker, predictors that otherwise would be masked by stronger predictors.

#### Variable importance

Random forests provide a very useful evaluation of the relative variable importance for prediction. The importance analysis is done by randomly resampling (scrambling) one variable at a time. This is equivalent to replacing the variable with noise. Model predictive ability is then evaluated in terms of how much mean squared error is increased. Replacing a strong predictor with noise will have a large effect on predictive ability. It may also happen that model prediction is slightly increased, which means that the original variable did not contribute to the prediction more than just a random noise. The largest improvement in prediction among all variables is used as a measure of prediction by error, and this amount is considered as Null improvement. To be of importance, a variable has to have importance larger than this Null. When variable importance is plotted for each of the variables, this Null value is usually marked to show which variables predict better than noise.

#### Predictive accuracy

We used the Receiver Operating Characteristic (ROC) curve as a tool to measure accuracy. A ROC curve is a plot of true positive rate versus false positive. The area under the curve (AUC) (a C-statistic) is equal to 0.5 if the model does not have any discriminating power and the area is equal to 1 when the model predicts the new outcomes perfectly well. A reliable and valid AUC estimate can be interpreted as the probability that the model will assign a higher score to a randomly chosen positive example than to a randomly chosen negative example.

## Results

### Selected sample characteristics

The sample sizes used in the analysis are summarized in Table 1. The prevalence of having ≥3 ED visits ranged from 4.0% in 2016 to 4.3% in 2017. The distributions of demographic variables (age groups, gender, race/ethnicity, education, marital status, and insurance status) were relatively consistent across the 4 survey years (2015–2018).

**Table 1:** Summary of sociodemographic variables and ED visits in the past 12 months among adults in the national sample of the NSDUH

Sample Characteristic	Training Dataset: 2017		Validation Dataset: 2018		Additional Validation Dataset: 2015		Additional Validation Dataset: 2016	
	Count	Weighted %	Count	Weighted %	Count	Weighted %	Count	Weighted %
AGE GROUP								
18–20	4992	5.2	5125	5.2	5306	5.4	4912	5.2
21–25	8848	8.7	8512	8.5	9247	9.0	8748	9.0
25–29	3989	7.3	3868	7.1	4081	7.1	3948	7.2
30–34	4797	8.7	4926	9.0	5003	8.7	4803	8.7
35–49	11,214	24.7	11,688	24.6	11,169	24.9	11,361	24.8
50–64	4997	25.3	4938	25.0	5157	25.7	5241	25.5
65 or Older	3717	20.2	3969	20.7	3598	19.2	3612	19.7
GENDER								
Male	19,987	48.3	20,169	48.3	19,828	48.2	19,853	48.2
Female	22,567	51.7	22,857	51.7	23,733	51.8	22,772	51.8
RACE/ETHNICITY								
Non-Hispanic White	25,870	63.8	25,834	63.4	26,025	64.7	25,969	64.4
Non-Hispanic Black	5230	11.9	5400	11.9	5502	11.8	5474	11.8
Non-Hispanic Other	4286	8.2	4327	8.4	4386	7.9	4110	8.1
Hispanic	7168	16.1	7465	16.3	7648	15.6	7072	15.7
EDUCATION								
Completed 6th grade or less	632	2.0	678	2.3	798	2.4	735	2.5
Completed 7th – 9th grades	1228	3.2	1219	3.1	1521	3.9	1216	3.3
Completed 10th – 12th grades	3535	7.0	3540	6.9	3980	7.8	3538	7.0
Completed High School or Higher	37,159	87.7	37,589	87.7	37,262	85.9	37,136	87.1
FAMILY INCOME								
< $20,000	8370	16.1	8118	15.7	9703	17.9	8939	17.0
$20,000–$49,999	13,321	29.5	13,139	29.4	14,015	30.0	13,493	30.0
$50,000–$74,999	6704	15.9	6672	15.5	6770	16.6	6543	15.9
≥ $75,000	14,159	38.5	15,097	39.3	13,073	35.5	13,650	37.1
MARITAL STATUS								
Married	17,653	51.9	17,929	51.6	18,046	52.7	17,471	51.7
Never Married	19,235	28.9	19,286	28.9	19,053	27.1	19,112	28.5
Divorced, Separated, or Widowed	5666	19.2	5811	19.5	6462	20.2	6042	19.8
INSURANCE STATUS								
Covered by Health Insurance	37,790	90.5	38,097	90.1	38,104	89.4	37,755	90.5
Not Covered	4764	9.5	4929	9.9	5457	10.6	4870	9.5
EMERGENCY DEPT. VISITS*								
< 3 Visits	39,707	94.0	40,185	94.2	40,600	94.2	39,726	94.1
3+ Visits	2013	4.2	1942	4.0	2127	4.0	2006	4.0
Missing data	834	1.8	899	1.8	834	1.8	893	1.9

*Missing data on ED visit were excluded from the analysis

### Predictive accuracy

All models, including the model with all 46 variables as main effects, performed relatively similar to each other when compared on predictive ability on the test set (Table 2). The stepwise regression model achieved AUC of 0.8 on training and validation datasets. The estimates of AUC were quite tight: bootstrap-based estimates of the standard deviation of AUCs for training and test sets was less than 0.01. As expected, a single tree model predicted the worst, in part because it was designed to provide interpretable logical structure that would provide clustering of the sample according to the probability of recurrent ED visits.

**Table 2:** Model fitting and predictive performance: ≥3 emergency department visits/year

Analysis sample	Training Dataset: 2017	Validation Dataset: 2018	Additional Validation Dataset: 2015	Additional Validation Dataset: 2016
Model	AUC	AUC	AUC	AUC
All variables regression	0.80	0.79	0.79	0.79
LASSO	0.80	0.79	0.79	0.79
Stepwise regression	0.80	0.79	0.79	0.79
Single tree	0.76	0.75	0.75	0.76
Stepwise regression with complex interactions	0.80	0.80	0.79	0.79
Random forest	0.88	0.79	0.79	0.79

Note: All bootstrap-estimated standard deviations of the AUCs were < 0.01. Because the model based on 2017 data has shown stability over the years, we have combined data from 2015 to 2018 to produce more stable estimates of odds ratios associated with multiple visits to the ED. In Table 3, we present the odds ratios and variable ranking from stepwise and forest models for 2015–2018 data

*AUC* Area under the curve, *LASSO* Least absolute shrinkage and selection operator, *ED* Emergency department

Although regression models showed similar performance, they differed in the number of variables from 46 in the full model to 28 in the stepwise regression. These results suggest that the predictions and the factors associated with frequent visits to ED are quite robust and also that some variables are more important for prediction than others.

**Fig. 1:**
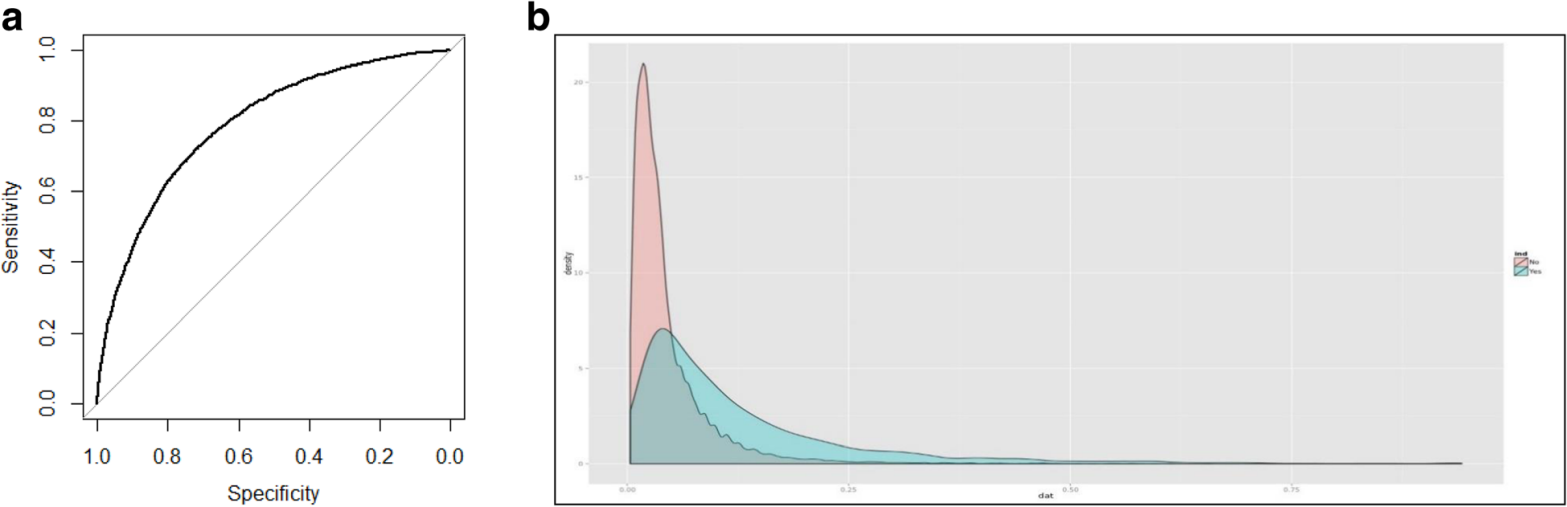
**a.** ROC Curve Representing 0.79 Predictive Capability of the LASSO Regression Model on Validation Data from 2018. **b**. A Double Histogram Illustrating Positive Predictive Value (PPV) Depending on the Predicted Score. Most individuals with the predicted probability over 0.25 are frequent (3+ times per year) visitors to ED department

Because the prevalence of multiple visits was small, for the majority of individuals the probability of multiple visits remained < 0.5 (i.e., a person was more likely not going to be a frequent ED user). However, for those who score over 0.20 (4 times over the mean of 0.05) the positive predictive value (PPV) was very high, over 0.98, i.e., out of those who scored positive a chance that they are frequent ED user is 0.98. For a small percentage of subjects who scored over 0.5 (more likely to be a frequent ED user than not) the PPV was over 0.999, which made the model a good screening test to identify extreme cases. A plot of a ROC curve (Fig. 1A) is another illustration of the balance between sensitivity and specificity in the best LASSO model, and in Fig. 1B we illustrate that the tail of the probability distributions contains mostly frequent ED users.

### Variable importance and consistency

#### Tree variable

A decision tree had a restriction of 400 observations per node and resulted in 14 nodes. This tree variable has a predictive AUC of 0.76 when predicting the 2018 data, which captures the essence of the interactions between the main variables.

Using the training data to produce the decision tree, the top interaction most related to multiple visits were between the self-rated health and having asthma. With respondents indicating fair/poor self-rated health combined with having asthma produced an estimate of 25% chance of multiple visits (more than five times the average). Those with fair/poor self-rated health with no asthma, but who were nervous all or most of the time, had 19% chance of multiple visits. Those with fair/poor self-rated health with no asthma and who were not nervous most of the time, but who used illicit drugs were the third largest category with 16% chance. Those with excellent/very good/good health, with income <$50,000, being restless most or all the time, and being female had 12% chance of multiple visits. Nicotine dependence, and African American race were also important factors that modified a group with excellent/very good/good health, income <$50,000 and not being restless most or all times. Among factors that decreased the chances of multiple visits were higher income and older age.

#### Variable selection and importance

Using 2015–2018 data, random forest-based variable importance was assessed. We ranked variables based on their permutation-based importance and compared to the variable list ranked by the formally calculated *p*-values in a stepwise regression. These nominal p-values would have been true p-values if the resulting regression model was a hypothesis-driven single analysis model. These quantities ignore multiple testing and the arbitrary selection of the training set, nevertheless, they are useful to evaluate the consistency of variables’ *ranks* in different modeling approaches (Table 3).

**Table 3:** First 25 Predictors of ≥3 emergency department visits in the past 12 months based on 2015–2018 data

Variable Name	Reference Cell	Odds Ratio in a stepwise regression	Order in Stepwise regression based on formal ***p***-value of first category occurrence	Order in Random Forest Model Importance
Fair/Poor Health	Excellent/Very Good/Good Self-Reported Heath	2.63	1	1
Income <$25,000	Income = > $75,000	2.53	2	2
Female Gender	Male	1.62	9	3
Asthma (Current)	No Asthma	1.87	8	4
All/Most of the Time of the Time Feeling Restless or Fidgety in Past Year	Some/Little/None of the Time	1.47	16	5
18–20 Years Old	Age 50–64 Years Old	2.30	4	6
Black/African American Race	White Race	1.91	7	7
Heart Condition in Past Year	No Heart Condition	2.30	5	8
All/Most of the Time of the Time Feeling Nervous in Past Year	Some/Little/None of the Time	1.28	26	9
Education Level: 10th–12th grades completed	6th grade or less completed	1.43	17	10
Diabetes (Ever)	No	1.62	10	11
Nicotine Dependence (Past Month)	No	1.38	19	12
Marital Status: Divorced, Separated, or Widowed	Married	1.16	33	13
COPD/Chronic Bronchitis (Ever)	No	1.48	15	14
Tobacco Use (Past Year)	No	1.29	23	15
Major Depressive Episode (Past Year)	No	1.23	25	16
No High Blood Pressure or Not Taking Medication for High Blood Pressure	Ever had High Blood Pressure or Currently Taking Medication	1.29	17	17
Illicit Drug Use (Past Year)	No	1.33	21	18
Alcohol Use (Past Year)	No	0.81	29	19
Illicit Drug Use Disorder (Past Year)	No	–	–	20
Sexually Transmitted Disease (Past Year)	No	1.58	11	21
Pain Reliever Misuse (Past Year)	No	1.29	24	22
Had Insurance Coverage	No Coverage	1.19	32	23
Pain Reliever Use Disorder (Past Year)	No	1.49	13	24
Population Density: Segment not in a CBSA	Segment is in a CBSA	1.25	28	25

Note: In random forest, variable importance is evaluated with respect to the variable as a whole, rather than a specific category. In fact, the variable categories are randomly “scrambled.” Thus, only one ranking value is issued for the variable. *COPD* Chronic obstructive pulmonary disease, *CBSA* Core Based Statistical Area

— The variable/category is not present in the top category list or was not selected by the stepwise algorithm

Table 3 shows the variable importance according to the random forest for the top 25 variables according to the ranking of the *p*-values in the stepwise regression model. The top 25 variables according to the stepwise regression model covered the top 20 variables from the random forest model, except for education level (ranked 11 in the random forest model) and marital status (ranked 14 in the random forest model).

The highest-ranking variable in the 2015–2018 random forest model was fair/poor self-rated health followed by low income. Other strong predictors include gender, currently having asthma, restlessness, having a heart condition in the past year, race, age, ever having diabetes, feeling nervous or fidgety, education level, and nicotine dependence.

A similar list of variables appeared when we ranked variables in multiple regressions. In evaluating the model results on the combined 2015–2018 data, the largest effect sizes with OR ≥ 1.8 estimated by LASSO were the following variables: fair/poor health (OR > 2.6), lower income (OR > 2.5), having a heart condition in the past year (OR > 2.4), HIV/AIDS (OR > 2.1), being of younger age (OR > 2.0), being of African American race (OR > 1.8), and having asthma (OR > 1.8). Female gender, which appeared near the top of the list for in the other models, was also a predictive variable in the LASSO model with an OR of > 1.6.

## Discussion

We developed and validated predictive models in a large sample that estimated probabilities for an individual to have three or more ED visits within a year. For a national household sample, the models showed good predictive ability AUC reaching 0.8. The model appears to be stable across the studied years that suggests the stability of the estimates as well as a persistent behavior with respect to visiting an ED. The most influential variable that showed a strong and consistent effect across models was self-rated personal health fair/poor rating was the best predictor of multiple ED visits. This result is consistent with past clinical research [29]. By construct, the most influential variables impact sensitivity/specificity at the overall population level. Such influential variables, however, can miss potential risk factors with high ORs but of low prevalence. If only few people have the given condition, their contribution to the population-level prediction can be small. We thus also considered the variables according to the size of their effects. One of such variables is cancer/malignancy of the larynx/windpipe or lung which produced an odds ratio of 2.6, but was not selected neither in the random forest nor in LASSO because of the relatively small sample. This effect became even more illustrative when we replicated the predictive process on data prior to 2015. For example, NSDUH datasets prior to 2015 contained variables on lung cancer and pancreatic diseases. When replicating the analysis on public use NSDUH 2014 data, the odds ratio estimates for lung cancer or pancreatic disease was large (> 4), however, because of a low prevalence (or small numbers of cases), their contribution to AUC improvement was small and they did not reach high statistical importance. At the same time, clinical importance could be high. That is, if a person happens to have a pancreatic disease, the chances of multiple visits to the ED could be highly increased.

Our findings are consistent with the ED use data from the Healthcare Cost and Utilization Project Nationwide ED sample, which shows that mood and anxiety disorders are among the leading contributors to ED visits and that younger adults and women have more ED visits than men and older adults [30]. The influential effect of nicotine dependence on multiple ED visits reaffirms a need to enhance smoking cessation, especially for African Americans and less-educated adults who are disproportionately affected by cigarette smoking [31, 32]. Our results also indicate that African Americans/blacks and less-educated adults have a high likelihood of multiple ED visits within a year, which may be related to their comparatively high levels of disparities in healthcare use and poor health status [33, 34]. The findings also highlight alcohol and illicit drug use as risk factors in multiple visits to ED.

These results have important implications for public health research. First, national healthcare reforms have shifted the healthcare model to a value-based model to address high costs and poor care concern in the U.S. [35]. Our findings indicate the need to identify effective care models to improve preventive services and continuity of care for less-educated adults, African Americans, and adults with tobacco use, anxiety, or depressive disorder. Second, the large national sample of the NSDUH provides the basis for testing robust validated models. Third, our findings have methodological implications. Variables with large effect sizes but low on predictive importance are indicative of the presence of low prevalence but high-risk clusters in the population. We thus distinguish between individualized and population-based predictions. We conducted post-hoc analyses in which we used the subsets of the pulled data to show that using a subset of the training set larger than 30,000 observations produced very similar population-based predictive models in terms of variable selection, effect size, and predictive performance to the original analysis that used about 60,000 observations as a training set. However, in order to identify small high-risk clusters large sample sizes provide necessary power and multiple years of the data could be combined to identify the specific effect sizes more precisely.

### Limitations and future work

Predictive models based on nonclinical assessments from the NSDUH have predictive limitations. Although the list of medical conditions used in NSDUH is large, it is not complete and might miss some of the potentially important predictors. More research on large samples that includes a diverse set of measures is needed to improve predictive accuracy, especially at the identification of high-risk clusters.

Survey data from NSDUH contain analysis weights to produce unbiased estimates of population prevalence. Use of these weights may have the potential for development of better predictive models when the model parameters are unbiased. Although the use of analysis weights for regression methods is standard, methodology for the use of survey weights in tree-based models has not been well developed beyond initial assessment of sensitivity to weights [36]. We conducted sensitivity analyses to compare predictive performance of weighted and unweighted regression models and did not find any improvements in the AUC or the selection of the top predictors. LASSO models do not produce *p*-values, because standard errors are not very meaningful for strongly biased estimates such as arise from penalized estimation methods [37, 38]. Nevertheless, LASSO models are state of the art and have been broadly used to select the best subset of predictive variables [39].

## Conclusions

A validated predictive model of frequent (three or more) ED visits within a year indicated that besides medical conditions, such as asthma, heart condition/disease, COPD, and diabetes, behavioral and mental health factors including lower self-rated health, being nervous or restless/fidgety, low income, low educational level, nicotine dependence, and depressive/anxiety signs or symptoms are among the most influential factors for multiple ED visits. These findings reveal the need to address behavioral and mental health contributors to ED visits and reinforce the importance of developing integrated care models in primary care settings to improve mental health for medically vulnerable patients. Although the list of medical conditions used in NSDUH is large, it is not complete and might miss some of the potentially important predictors. The remarkable consistency of predictive ability across the years assures that the results are stable and that the behavior of the subjects with respect to ED visits did not change much over the last several years. Presented modeling approach can be broadly applied to national and other large surveys.

## Supplementary Information

**Additional file 1: Table A1.** Sociodemographic characteristics, substance use behaviors, mental health issues, and prevalence of ED visits in the past 12 months among adults in the national sample of the NSDUH.

## Data Availability

The datasets used and analyzed are in public domain and are also available from the corresponding author on reasonable request.

## Footnotes

1 Marital status was changed to ACASI in 2015 then, because of data quality issues, was moved back to CAPI in 2016-2018. Education also was administered with ACASI.

2 Past month pipe tobacco use is used in this measure because past year pipe tobacco use is not available.

3 Although all the pain reliever subtypes referenced in the NSDUH questionnaire are opioids, respondents could specify that they misused a non-opioid pain reliever (e.g., prescription-strength ibuprofen) [16].
