# Supplementary material for: Predictive model of multiple emergency department visits among adults: analysis of the data from the National Survey of Drug Use and Health (NSDUH)
Source: BMC Health Serv Res. 2021 Mar 25;21:280. doi: 10.1186/s12913-021-06221-w (PMC7995604; doi:10.1186/s12913-021-06221-w)
Supplement: Supplementary file 1 — Additional file 1: Table A1. Sociodemographic characteristics, substance use behaviors, mental health issues, and prevalence of ED visits in the past 12 months among adults in the national sample of the NSDUH. [file 12913_2021_6221_MOESM1_ESM.docx]

Table A1. Sociodemographic characteristics, substance use behaviors, mental health issues, and prevalence of ED visits in the past 12 months among adults in the national sample of the NSDUH

| **Sample**  **Characteristic** | **Training**  **Dataset: 2017** | | **Validation**  **Dataset: 2018** | | **Additional Validation Dataset: 2015** | | **Additional Validation Dataset: 2016** | |
| --- | --- | --- | --- | --- | --- | --- | --- | --- |
|  | **Count** | **Weighted %** | **Count** | **Weighted %** | **Count** | **Weighted %** | **Count** | **Weighted %** |
| AGE GROUP |  |  |  |  |  |  |  |  |
| 18-20 | 4,992 | 5.2 | 5,125 | 5.2 | 5,306 | 5.4 | 4,912 | 5.2 |
| 21-25 | 8,848 | 8.7 | 8,512 | 8.5 | 9,247 | 9.0 | 8,748 | 9.0 |
| 25-29 | 3,989 | 7.3 | 3,868 | 7.1 | 4,081 | 7.1 | 3,948 | 7.2 |
| 30-34 | 4,797 | 8.7 | 4,926 | 9.0 | 5,003 | 8.7 | 4,803 | 8.7 |
| 35-49 | 11,214 | 24.7 | 11,688 | 24.6 | 11,169 | 24.9 | 11,361 | 24.8 |
| 50-64 | 4,997 | 25.3 | 4,938 | 25.0 | 5,157 | 25.7 | 5,241 | 25.5 |
| 65 or Older | 3,717 | 20.2 | 3,969 | 20.7 | 3,598 | 19.2 | 3,612 | 19.7 |
| GENDER |  |  |  |  |  |  |  |  |
| Male | 19,987 | 48.3 | 20,169 | 48.3 | 19,828 | 48.2 | 19,853 | 48.2 |
| Female | 22,567 | 51.7 | 22,857 | 51.7 | 23,733 | 51.8 | 22,772 | 51.8 |
| RACE/ETHNICITY |  |  |  |  |  |  |  |  |
| Non-Hispanic White | 25,870 | 63.8 | 25,834 | 63.4 | 26,025 | 64.7 | 25,969 | 64.4 |
| Non-Hispanic Black | 5,230 | 11.9 | 5,400 | 11.9 | 5,502 | 11.8 | 5,474 | 11.8 |
| Non-Hispanic Other | 4,286 | 8.2 | 4,327 | 8.4 | 4,386 | 7.9 | 4,110 | 8.1 |
| Hispanic | 7,168 | 16.1 | 7,465 | 16.3 | 7,648 | 15.6 | 7,072 | 15.7 |
| EDUCATION |  |  |  |  |  |  |  |  |
| Completed 6^th^ grade or less | 632 | 2.0 | 678 | 2.3 | 798 | 2.4 | 735 | 2.5 |
| Completed 7^th^ – 9^th^ grades | 1,228 | 3.2 | 1,219 | 3.1 | 1,521 | 3.9 | 1,216 | 3.3 |
| Completed 10^th^ – 12^th^ grades | 3,535 | 7.0 | 3,540 | 6.9 | 3,980 | 7.8 | 3,538 | 7.0 |
| Completed High School or Higher | 37,159 | 87.8 | 37,589 | 87.7 | 37,262 | 85.9 | 37,136 | 87.1 |
| FAMILY INCOME |  |  |  |  |  |  |  |  |
| <$20,000 | 8,370 | 16.1 | 8,118 | 15.8 | 9,703 | 17.9 | 8,939 | 17.0 |
| $20,000-$49,999 | 13,321 | 29.5 | 13,139 | 29.4 | 14,015 | 30.0 | 13,493 | 30.0 |
| $50,000-$74,999 | 6,704 | 15.9 | 6,672 | 15.5 | 6,770 | 16.7 | 6,543 | 15.9 |
| ≥$75,000 | 14,159 | 38.5 | 15,097 | 39.4 | 13,073 | 35.5 | 13,650 | 37.1 |

| **Sample**  **Characteristic** | **Training**  **Dataset: 2017** | | **Validation**  **Dataset: 2018** | | **Additional Validation Dataset: 2015** | | **Additional Validation Dataset: 2016** | |
| --- | --- | --- | --- | --- | --- | --- | --- | --- |
|  | **Count** | **Weighted %** | **Count** | **Weighted %** | **Count** | **Weighted %** | **Count** | **Weighted %** |
| MARITAL STATUS |  |  |  |  |  |  |  |  |
| Married | 17,653 | 51.9 | 17,929 | 51.6 | 18,046 | 52.7 | 17,471 | 51.7 |
| Never Married | 19,235 | 28.9 | 19,286 | 28.9 | 19,053 | 27.1 | 19,112 | 28.5 |
| Divorced, Separated, or Widowed | 5,666 | 19.3 | 5,811 | 19.5 | 6,462 | 20.2 | 6,042 | 19.8 |
| INSURANCE STATUS |  |  |  |  |  |  |  |  |
| Covered by Health Insurance | 37,790 | 90.5 | 38,097 | 90.1 | 38,104 | 89.4 | 37,755 | 90.5 |
| Not Covered | 4,764 | 9.5 | 4,929 | 9.9 | 5,457 | 10.6 | 4,870 | 9.5 |
| POPULATION DENSITY |  |  |  |  |  |  |  |  |
| CBSA with 1 million or more persons | 17,857 | 54.3 | 18,415 | 54.0 | 18,831 | 54.0 | 18,116 | 53.7 |
| CBSA with fewer than 1 million persons | 21,202 | 39.8 | 21,231 | 40.2 | 21,468 | 40.3 | 21,273 | 40.5 |
| Segment not in a CBSA | 3,495 | 5.9 | 3,380 | 5.9 | 3,262 | 5.7 | 3,236 | 5.8 |
| SELF-RATED HEALTH |  |  |  |  |  |  |  |  |
| Excellent/Very Good/Good | 37,712 | 86.1 | 38,171 | 86.2 | 38,567 | 86.0 | 37,830 | 86.0 |
| Fair/Poor | 4,829 | 13.9 | 4,846 | 13.8 | 4,990 | 14.0 | 4,792 | 14.0 |
| Missing data | 13 | 0.0 | 9 | 0.0 | 4 | 0.0 | 3 | 0.0 |
| ASTHMA (CURRENT) |  |  |  |  |  |  |  |  |
| Yes | 2,997 | 6.6 | 2,905 | 6.1 | 3,070 | 6.1 | 2,957 | 6.0 |
| No | 39,124 | 92.4 | 39,652 | 92.9 | 40,096 | 93.2 | 39,222 | 93.0 |
| Missing data | 433 | 1.0 | 469 | 1.0 | 395 | 0.8 | 446 | 1.0 |
| CHRONIC BRONCHITIS OR COPD (EVER) |  |  |  |  |  |  |  |  |
| Yes | 1,260 | 4.6 | 1,161 | 4.1 | 1,324 | 4.4 | 1,261 | 4.3 |
| No | 40,889 | 94.5 | 41,423 | 95.0 | 41,864 | 94.9 | 40,948 | 94.8 |
| Missing data | 405 | 0.9 | 442 | 0.9 | 373 | 0.7 | 416 | 1.0 |
| LUNG CANCER (EVER) |  |  |  |  |  |  |  |  |
| Yes | 40 | 0.2 | 46 | 0.2 | 36 | 0.2 | 44 | 0.2 |
| No | 42,075 | 98.8 | 42,494 | 98.7 | 43,119 | 99.0 | 42,133 | 98.8 |
| Missing data | 439 | 1.0 | 486 | 1.1 | 406 | 0.8 | 448 | 1.1 |

| **Sample**  **Characteristic** | **Training**  **Dataset: 2017** | | **Validation**  **Dataset: 2018** | | **Additional Validation Dataset: 2015** | | **Additional Validation Dataset: 2016** | |
| --- | --- | --- | --- | --- | --- | --- | --- | --- |
|  | **Count** | **Weighted %** | **Count** | **Weighted %** | **Count** | **Weighted %** | **Count** | **Weighted %** |
| HEART CONDITION (PAST YEAR) |  |  |  |  |  |  |  |  |
| Yes | 1,462 | 5.7 | 1,564 | 5.7 | 1,420 | 5.2 | 1,397 | 5.4 |
| No | 40,673 | 93.3 | 41,006 | 93.3 | 41,757 | 94.0 | 40,807 | 93.6 |
| Missing data | 419 | 1.0 | 456 | 1.0 | 384 | 0.8 | 421 | 1.0 |
| HIGH BLOOD PRESSURE (EVER OR TAKING MEDICATION) |  |  |  |  |  |  |  |  |
| Yes | 5,178 | 19.4 | 5,281 | 19.2 | 5,375 | 19.9 | 5,274 | 19.7 |
| No | 36,971 | 79.7 | 37,303 | 79.8 | 37,813 | 79.4 | 36,935 | 79.3 |
| Missing data | 405 | 0.9 | 442 | 0.9 | 373 | 0.7 | 416 | 1.0 |
| CIRRHOSIS OF LIVER (EVER) |  |  |  |  |  |  |  |  |
| Yes | 65 | 0.2 | 85 | 0.4 | 69 | 0.3 | 88 | 0.4 |
| No | 42,084 | 98.8 | 42,499 | 98.7 | 43,119 | 99.0 | 42,121 | 98.7 |
| Missing data | 405 | 0.9 | 442 | 0.9 | 373 | 0.7 | 416 | 1.0 |
| HEPATITIS B OR C (EVER) |  |  |  |  |  |  |  |  |
| Yes | 395 | 1.2 | 360 | 1.3 | 379 | 1.3 | 405 | 1.5 |
| No | 41,754 | 97.9 | 42,224 | 97.8 | 42,809 | 98.0 | 41,804 | 97.6 |
| Missing data | 405 | 0.9 | 442 | 0.9 | 373 | 0.7 | 416 | 1.0 |
| DIABETES (EVER) |  |  |  |  |  |  |  |  |
| Yes | 2,793 | 10.1 | 3,001 | 11.0 | 2,961 | 10.6 | 2,839 | 10.4 |
| No | 39,356 | 89.0 | 39,583 | 88.1 | 40,227 | 88.7 | 39,370 | 88.7 |
| Missing data | 405 | 0.9 | 442 | 0.9 | 373 | 0.7 | 416 | 1.0 |
| HIV/AIDS (EVER) |  |  |  |  |  |  |  |  |
| Yes | 84 | 0.2 | 92 | 0.3 | 78 | 0.2 | 83 | 0.2 |
| No | 42,065 | 98.9 | 42,492 | 98.8 | 43,110 | 99.1 | 42,126 | 98.9 |
| Missing data | 405 | 0.9 | 442 | 0.9 | 373 | 0.7 | 416 | 1.0 |

| **Sample**  **Characteristic** | **Training**  **Dataset: 2017** | | **Validation**  **Dataset: 2018** | | **Additional Validation Dataset: 2015** | | **Additional Validation Dataset: 2016** | |
| --- | --- | --- | --- | --- | --- | --- | --- | --- |
|  | **Count** | **Weighted %** | **Count** | **Weighted %** | **Count** | **Weighted %** | **Count** | **Weighted %** |
| ANY STD (PAST YEAR) |  |  |  |  |  |  |  |  |
| Yes | 1,059 | 2.0 | 1,099 | 2.1 | 1,166 | 2.0 | 1,066 | 2.0 |
| No | 41,269 | 97.6 | 41,667 | 97.4 | 42,210 | 97.7 | 41,327 | 97.5 |
| Missing data | 226 | 0.4 | 260 | 0.5 | 185 | 0.3 | 232 | 0.5 |
| HOW OFTEN FELT NERVOUS |  |  |  |  |  |  |  |  |
| All/Most of the Time | 7,430 | 13.1 | 7,608 | 13.3 | 6,887 | 12.4 | 7,023 | 12.7 |
| Some/A Little/None of the Time | 34,853 | 86.2 | 35,100 | 86.0 | 36,394 | 87.1 | 35,326 | 86.7 |
| Missing data | 271 | 0.7 | 318 | 0.7 | 280 | 0.6 | 276 | 0.6 |
| HOW OFTEN FELT RESTLESS |  |  |  |  |  |  |  |  |
| All/Most of the Time | 5,802 | 9.7 | 5,893 | 9.9 | 5,351 | 9.1 | 5,414 | 9.3 |
| Some/A Little/None of the Time | 36,456 | 89.6 | 36,772 | 89.3 | 37,903 | 90.3 | 36,895 | 90.0 |
| Missing data | 296 | 0.7 | 361 | 0.8 | 307 | 0.6 | 316 | 0.7 |
| MAJOR DEPRESSIVE EPISODE (PAST YEAR) |  |  |  |  |  |  |  |  |
| Yes | 3,949 | 7.1 | 3,953 | 7.0 | 3,571 | 6.7 | 3,590 | 6.6 |
| No | 38,117 | 91.8 | 38,524 | 91.8 | 39,511 | 92.3 | 38,544 | 92.3 |
| Missing data | 488 | 1.0 | 549 | 1.1 | 479 | 1.0 | 491 | 1.2 |
| ALCOHOL USE (PAST YEAR) |  |  |  |  |  |  |  |  |
| Yes | 31,057 | 70.1 | 31,206 | 69.9 | 31,887 | 70.0 | 31,007 | 69.4 |
| No | 11,497 | 29.9 | 11,820 | 30.1 | 11,674 | 30.1 | 11,618 | 30.6 |
| TOBACCO USE (PAST YEAR) |  |  |  |  |  |  |  |  |
| Yes | 14,964 | 29.2 | 14,434 | 28.6 | 16,076 | 31.2 | 15,263 | 30.4 |
| No | 27,590 | 70.8 | 28,592 | 71.4 | 27,485 | 68.8 | 27,362 | 69.6 |

| **Sample**  **Characteristic** | **Training**  **Dataset: 2017** | | **Validation**  **Dataset: 2018** | | **Additional Validation Dataset: 2015** | | **Additional Validation Dataset: 2016** | |
| --- | --- | --- | --- | --- | --- | --- | --- | --- |
|  | **Count** | **Weighted %** | **Count** | **Weighted %** | **Count** | **Weighted %** | **Count** | **Weighted %** |
| ILLICIT DRUG USE (PAST YEAR) |  |  |  |  |  |  |  |  |
| Yes | 10,845 | 19.2 | 11,132 | 19.8 | 10,417 | 18.0 | 10,460 | 18.3 |
| No | 31,709 | 80.9 | 31,894 | 80.2 | 33,144 | 82.1 | 32,165 | 81.7 |
| MARIJUANA USE (PAST YEAR) |  |  |  |  |  |  |  |  |
| Yes | 9,024 | 15.2 | 9,509 | 16.3 | 8,292 | 13.6 | 8,564 | 14.2 |
| No | 33,530 | 84.8 | 33,517 | 83.7 | 35,269 | 86.4 | 34,061 | 85.9 |
| INHALANT USE (PAST YEAR) |  |  |  |  |  |  |  |  |
| Yes | 332 | 0.5 | 312 | 0.6 | 310 | 0.5 | 291 | 0.5 |
| No | 42,222 | 99.5 | 42,714 | 99.4 | 43,251 | 99.5 | 42,334 | 99.6 |
| COCAINE USE (PAST YEAR) |  |  |  |  |  |  |  |  |
| Yes | 1,377 | 2.4 | 1,334 | 2.2 | 1,171 | 1.9 | 1,232 | 2.0 |
| No | 41,177 | 97.7 | 41,692 | 97.8 | 42,390 | 98.1 | 41,393 | 98.0 |
| HALLUCINOGEN USE (PAST YEAR) |  |  |  |  |  |  |  |  |
| Yes | 1,308 | 1.9 | 1,339 | 2.1 | 1,327 | 1.8 | 1,284 | 1.8 |
| No | 41,246 | 98.2 | 41,687 | 97.9 | 42,234 | 98.2 | 41,341 | 98.2 |
| HEROIN USE (PAST YEAR) |  |  |  |  |  |  |  |  |
| Yes | 216 | 0.4 | 193 | 0.3 | 211 | 0.3 | 240 | 0.4 |
| No | 42,338 | 99.6 | 42,833 | 99.7 | 43,350 | 99.7 | 42,385 | 99.6 |
| PRESCRIPTION RELIEVER MISUSE (PAST YEAR) |  |  |  |  |  |  |  |  |
| Yes | 2,269 | 4.1 | 1,948 | 3.7 | 2,672 | 4.7 | 2,311 | 4.4 |
| No | 40,285 | 95.9 | 41,078 | 96.3 | 40,889 | 95.3 | 40,314 | 95.6 |

| **Sample**  **Characteristic** | **Training**  **Dataset: 2017** | | **Validation**  **Dataset: 2018** | | **Additional Validation Dataset: 2015** | | **Additional Validation Dataset: 2016** | |
| --- | --- | --- | --- | --- | --- | --- | --- | --- |
|  | **Count** | **Weighted %** | **Count** | **Weighted %** | **Count** | **Weighted %** | **Count** | **Weighted %** |
| PRESCRIPTION STIMULANT MISUSE (PAST YEAR) |  |  |  |  |  |  |  |  |
| Yes | 1,479 | 2.2 | 1,306 | 1.9 | 1,480 | 2.0 | 1,499 | 2.2 |
| No | 41,075 | 97.8 | 41,720 | 98.1 | 42,081 | 98.0 | 41,126 | 97.8 |
| PRESCRIPTION SEDATIVES MISUSE (PAST YEAR) |  |  |  |  |  |  |  |  |
| Yes | 236 | 0.5 | 207 | 0.4 | 285 | 0.6 | 274 | 0.6 |
| No | 42,318 | 99.5 | 42,819 | 99.6 | 43,276 | 99.4 | 42,351 | 99.4 |
| PRESCRIPTION TRAQUILIZERS MISUSE (PAST YEAR) |  |  |  |  |  |  |  |  |
| Yes | 1,324 | 2.3 | 1,212 | 2.2 | 1,375 | 2.4 | 1,335 | 2.3 |
| No | 41,230 | 97.7 | 41,814 | 97.8 | 42,186 | 97.7 | 41,290 | 97.7 |
| ALCOHOL USE DISORDER (PAST YEAR) |  |  |  |  |  |  |  |  |
| Yes | 3,202 | 5.7 | 3,139 | 5.7 | 3,431 | 6.3 | 3,290 | 6.1 |
| No | 39,352 | 94.3 | 39,887 | 94.3 | 40,130 | 93.7 | 39,335 | 94.0 |
| NICOTINE DEPENDENCE (PAST YEAR) |  |  |  |  |  |  |  |  |
| Yes | 5,259 | 10.9 | 4,924 | 10.6 | 5,789 | 11.9 | 5,368 | 11.8 |
| No | 37,295 | 89.1 | 38,102 | 89.4 | 37,772 | 88.1 | 37,257 | 88.3 |
| ILLCIT DRUG USE DISORDER (PAST YEAR) |  |  |  |  |  |  |  |  |
| Yes | 1,814 | 2.7 | 1,820 | 3.0 | 1,767 | 2.8 | 1,719 | 2.7 |
| No | 40,740 | 97.3 | 41,206 | 97.0 | 41,794 | 97.2 | 40,906 | 97.3 |
| MARIJUANA USE DISORDER (PAST YEAR) |  |  |  |  |  |  |  |  |
| Yes | 1,026 | 1.4 | 1,115 | 1.6 | 1,001 | 1.4 | 974 | 1.4 |
| No | 41,528 | 98.6 | 41,911 | 98.4 | 42,560 | 98.6 | 41,651 | 98.6 |
| INHALANT USE DISORDER (PAST YEAR) |  |  |  |  |  |  |  |  |
| Yes | 25 | 0.0 | 20 | 0.0 | 16 | 0.0 | 14 | 0.0 |
| No | 42,529 | 100.0 | 43,006 | 100.0 | 43,545 | 100.0 | 42,611 | 100.0 |
| COCAINE USE DISORDER (PAST YEAR) |  |  |  |  |  |  |  |  |
| Yes | 218 | 0.4 | 215 | 0.4 | 176 | 0.3 | 191 | 0.4 |
| No | 42,336 | 99.6 | 42,811 | 99.6 | 43,385 | 99.7 | 42,434 | 99.6 |
| HALLUCINOGEN USE DISORDER (PAST YEAR) |  |  |  |  |  |  |  |  |
| Yes | 74 | 0.1 | 79 | 0.1 | 76 | 0.1 | 85 | 0.1 |
| No | 42,480 | 99.9 | 42,947 | 99.9 | 43,485 | 99.9 | 42,540 | 99.9 |
| HEROIN USE DISORDER (PAST YEAR) |  |  |  |  |  |  |  |  |
| Yes | 165 | 0.3 | 122 | 0.2 | 148 | 0.2 | 170 | 0.3 |
| No | 42,389 | 99.7 | 42,904 | 99.8 | 43,413 | 99.8 | 42,455 | 99.7 |
| PRESCRIPTION PAIN RELIEVER USE DISORDER (PAST YEAR) |  |  |  |  |  |  |  |  |
| Yes | 373 | 0.6 | 314 | 0.6 | 446 | 0.8 | 364 | 0.7 |
| No | 42,181 | 99.4 | 42,712 | 99.4 | 43,115 | 99.2 | 42,261 | 99.3 |
| PRESCRIPTION STIMULANT USE DISORDER (PAST YEAR) |  |  |  |  |  |  |  |  |
| Yes | 144 | 0.2 | 127 | 0.2 | 116 | 0.2 | 130 | 0.2 |
| No | 42,410 | 99.8 | 42,899 | 99.8 | 43,445 | 99.8 | 42,495 | 99.8 |
| PRESCRIPTION SEDATIVE USE DISORDER (PAST YEAR) |  |  |  |  |  |  |  |  |
| Yes | 34 | 0.1 | 27 | 0.0 | 33 | 0.1 | 31 | 0.1 |
| No | 42,520 | 99.9 | 42,999 | 100.0 | 43,528 | 100.0 | 42,594 | 99.9 |
| PRESCRIPTION TRANQUILIZER USE DISORDER (PAST YEAR) |  |  |  |  |  |  |  |  |
| Yes | 163 | 0.3 | 157 | 0.2 | 143 | 0.2 | 137 | 0.2 |
| No | 42,391 | 99.7 | 42,869 | 99.8 | 43,418 | 99.8 | 42,488 | 99.8 |
| EMERGENCY DEPARTMENT VISITS |  |  |  |  |  |  |  |  |
| <3 Visits | 39,707 | 94.0 | 40,185 | 94.2 | 40,600 | 94.2 | 39,726 | 94.1 |
| 3+ Visits | 2,013 | 4.2 | 1,942 | 4.0 | 2,127 | 4.0 | 2,006 | 4.0 |
| Missing data | 834 | 1.8 | 899 | 1.8 | 834 | 1.8 | 893 | 1.9 |

COPD: Chronic obstructive pulmonary disease; CBSA = Core Based Statistical Area

Source: National Survey on Drug Use and Health (NSDUH) public use files from 2015, 2016, 2017, and 2018.
